# Emphysema quantification using chest CT: influence of radiation dose reduction and reconstruction technique

**DOI:** 10.1186/s41747-018-0064-3

**Published:** 2018-11-07

**Authors:** Annemarie M. den Harder, Erwin de Boer, Suzanne J. Lagerweij, Martijn F. Boomsma, Arnold M. R. Schilham, Martin J. Willemink, Julien Milles, Tim Leiner, Ricardo P. J. Budde, Pim A. de Jong

**Affiliations:** 1 Department of Radiology, University Medical Center Utrecht, Utrecht, The Netherlands; 2 Department of Radiology, Isala hospital, Zwolle, The Netherlands; 3 Philips Healthcare, Best, The Netherlands; 4 Department of Radiology, Erasmus Medical Center, Rotterdam, The Netherlands

**Keywords:** Densitometry, Emphysema, Radiation dosage, Thorax, Tomography (x-ray computed)

## Abstract

**Background:**

Computed tomography *(*CT) emphysema quantification is affected by both radiation dose (i.e. image noise) and reconstruction technique. At reduced dose, filtered back projection (FBP) results in an overestimation of the amount of emphysema due to higher noise levels, while the use of iterative reconstruction (IR) can result in an underestimation due to reduced noise. The objective of this study was to determine the influence of dose reduction and hybrid IR (HIR) or model-based IR (MIR) on CT emphysema quantification.

**Methods:**

Twenty-two patients underwent inspiratory chest CT scan at routine radiation dose and at 45%, 60% and 75% reduced radiation dose. Acquisitions were reconstructed with FBP, HIR and MIR. Emphysema was quantified using the 15th percentile of the attenuation curve and the percentage of voxels below -950 HU. To determine whether the use of a different percentile or HU threshold is more accurate at reduced dose levels and with IR, additional measurements were performed using different percentiles and HU thresholds to determine the optimal combination.

**Results:**

Dose reduction resulted in a significant overestimation of emphysema, while HIR and MIR resulted in an underestimation. Lower HU thresholds with FBP at reduced dose and higher HU thresholds with HIR and MIR resulted in emphysema percentages comparable to the reference. The 15th percentile quantification method showed similar results as the HU threshold method.

**Conclusions:**

This within-patients study showed that CT emphysema quantification is significantly affected by dose reduction and IR. This can potentially be solved by adapting commonly used thresholds.

**Electronic supplementary material:**

The online version of this article (10.1186/s41747-018-0064-3) contains supplementary material, which is available to authorized users.

## Key points

- Dose reduction resulted in a significant CT overestimation of emphysema, while iterative reconstruction resulted in a significant underestimation
- This can potentially be solved by adapting the commonly used densitometry thresholds
- The maximal intraclass correlation coefficient between reduced dose and the reference standard was achieved at 75% reduced dose with hybrid iterative reconstruction

## Background

Chest computed tomography (CT) offers the possibility of quantifying the amount of emphysema. The number of chest CT acquisitions is expected to increase due to the favourable results of the National Lung Screening Trial [1] and the interest in subtyping chronic obstructive pulmonary disease (COPD) patients [2]. Additional quantification of emphysema on screening CT acquisitions will therefore likely gain importance. Furthermore, this additional information may also contribute to optimisation of the benefits and cost-effectiveness of CT screening [3]. CT can be used to both identify patients with emphysema as well as to monitor progression in patients with COPD. Although emphysema is traditionally a pathology-based diagnosis [4, 5], CT densitometry of the lungs has demonstrated it to be associated with airflow obstruction, forced expiratory volume in 1 s and severity according to the Global initiative for chronic Obstructive Lung Disease (GOLD) criteria [6–8]. CT densitometry is based on either the 15th percentile of the attenuation curve or the percentage of voxels below -950 HU because those parameters show the strongest correlation with microscopic and macroscopic emphysema findings [8, 9]. Although pulmonary function tests measure limitation of airflow, they are not able to differentiate between airway obstruction and emphysematous destruction. CT, on the other hand, provides in vivo information about pathological changes and allows for differentiation between airway obstruction and emphysematous destruction [10].

The increasing number of chest CT scans has urged the importance of radiation dose reduction. However, dose reduction leads to higher noise levels, especially when images are reconstructed using conventional filtered back projection (FBP). Therefore, several iterative reconstruction (IR) techniques were developed to reduce image noise [11, 12]. Recent studies showed that the radiation dose of unenhanced chest CT can be reduced to sub-millisievert dose levels when IR is applied [13].

It is known that CT emphysema quantification is affected by both radiation dose (i.e. image noise) and reconstruction technique. At reduced dose, FBP results in an overestimation of the amount of emphysema due to higher noise levels, while the use of IR can result in an underestimation due to reduced noise [14–16].

The primary aim of the current study was to determine the effect of both dose reduction and IR on CT emphysema quantification using a within-patients study design. The secondary aim was to investigate whether adapting CT densitometry thresholds is a valid way to correct for over- or underestimation at reduced dose and with IR.

## Methods

### Patients

This prospective study was approved by the local Institutional Review Board (NL46146.041.13) and all study participants provided written informed consent. Patients aged ≥ 50 years scheduled for follow-up of ≥ 1 known small pulmonary nodules were eligible for inclusion. The influence of dose reduction and IR on pulmonary nodule volume and computer-aided detection of pulmonary nodules was previously investigated in the same study population [17, 18].

### Image acquisition

Image acquisition was performed on a 256-slice CT system (Brilliance iCT; Philips Healthcare, Best, The Netherlands). An unenhanced chest CT was acquired during inspiration. The routine dose acquisition was performed with a tube voltage of 100 kVp (body weight < 80 kg) or 120 kV (body weight ≥ 80 kg). The tube current-time product was 60 mAs at routine dose and subsequently decreased to 33, 24 and 15 mAs to achieve 45%, 60% and 75% dose reduction, respectively. All four acquisitions were acquired consecutively in a single session. Automatic exposure control was off. Images were reconstructed at a slice thickness of 2 mm with FBP, hybrid IR (HIR; iDose level 4, Philips Healthcare, Best, The Netherlands) and model-based IR (MIR; IMR level 2, Philips Healthcare, Best, The Netherlands). Kernel filter C was used for both FBP and HIR. MIR is a more advanced reconstruction technique with different kernels; therefore, the vendor-recommended kernel filter Body Routine was used for MIR. The volume CT dose index (CTDI_vol_) and dose-length product (DLP) of each acquisition was recorded. The effective dose was calculated by multiplying the DLP with a conversion factor of 0.0144 (100 kVp) or 0.0145 (120 kVp) [19].

### Emphysema quantification

**Fig. 1:**
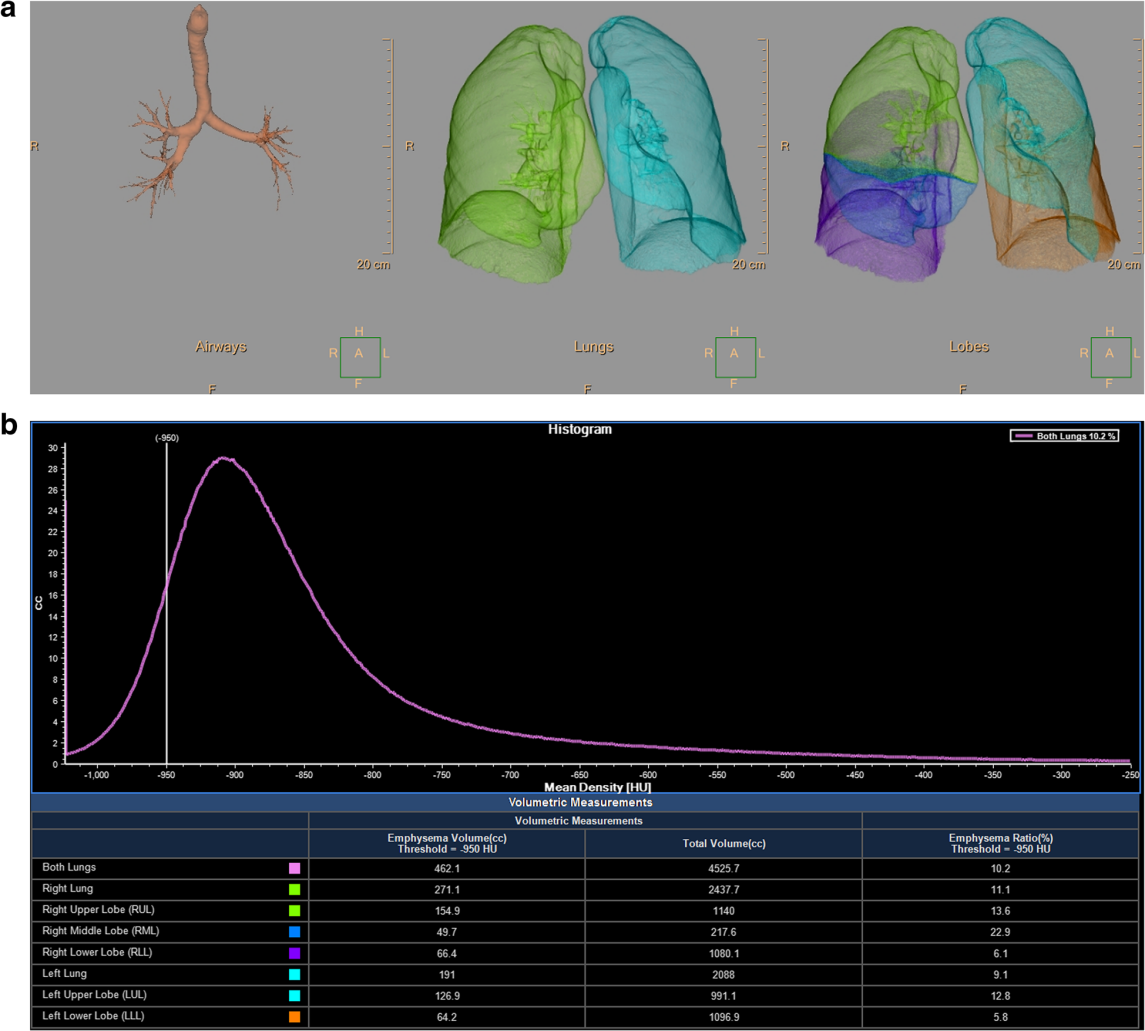
Example of the semi-automatic software which was used for emphysema quantification. First, the airways, lungs and lung lobes are segmented (**a**). Subsequently, a histogram is made which displays the number of voxels with a certain density (**b**). In this example the percentage of voxels below -950 HU is displayed

Semi-automatic commercially available software (IntelliSpace version 8, COPD tool, Philips Healthcare, Best, The Netherlands) was used for emphysema quantification. The noise reduction option in the software was not used. The software segments airways first, followed by the lungs and finally the different lobes. No manual segmentation was needed. Subsequently, a histogram (attenuation curve) is made which displays the number of voxels with a certain density (Fig. 1). Emphysema can be quantified by using either a percentile of the attenuation curve or the percentage of voxels below a certain HU value. On the routine dose acquisition reconstructed with FBP, emphysema was defined as a HU value which describes the lowest 15% of the segmented lungs (perc_15_). Furthermore, the percentage of voxels with a HU value of -950 HU or lower (percentage emphysema) was calculated. To determine whether the use of a different percentile or HU threshold is more accurate at reduced dose levels and with IR, additional measurements were performed as follows (1 percentage and 10 HU increments):

- reduced dose FBP: perc_8_ – perc_35_ and -960 HU – -1010 HU
- HIR:perc_1_ – perc_25_ and -880 HU – -960 HU
- MIR: perc_1_ – perc_20_ and -880 HU – -960 HU

### Objective image quality

A region of interest was placed in the ascending aorta at the level of the tracheal bifurcation and in the subcutaneous fat dorsal of the infraspinatus muscle. The noise was defined as the standard deviation of the region of interest and the contrast-to-noise ratio (CNR) was calculated using the following formula:

$$ \mathrm{CNR}=\frac{\mathrm{Mean}\ \left(\mathrm{Aorta}\right)-\mathrm{Mean}\ \left(\mathrm{Fat}\right)}{\sqrt{\frac{1}{2}\ast \left(\mathrm{SD}\ {\left(\mathrm{Aorta}\right)}^2+\mathrm{SD}{\left(\mathrm{Fat}\right)}^2\right)}} $$

### Statistics

Statistical analysis was performed using SPSS version 21 (SPSS Inc., Chicago, IL, USA). The routine dose acquisition reconstructed with FBP was used as the reference standard. The Friedman test was used to compare the reconstructions at each dose level to FBP and post-hoc analyses were performed with the Wilcoxon signed rank test. A *p* value < 0.05 was considered significant for the Friedman test, while a Bonferroni corrected *p* value of 0.017 (0.05/3 reconstructions) was used for the Wilcoxon test. The intraclass correlation coefficient (ICC; two-way mixed, absolute agreement, single measures) was used to compare reduced dose and iterative reconstruction to the reference standard. For each dose level and reconstruction technique, the optimal adapted threshold for emphysema quantification was determined. The optimal adapted threshold was also compared to the reference standard using Bland–Altman plots. Results are displayed as median (interquartile range) unless specified otherwise.

## Results

Twenty-two patients were included. Half of the patients (*n* = 11) were female. Ten patients (46%) were scanned with 100 kVp (< 80 kg) and twelve patients (54%) with 120 kVp (≥ 80 kg). The median height of the patients was 169 cm (163–176 cm) and the median weight was 83 kg (74–92 kg) resulting in a body mass index of 28.6 kg/m^2^ (26.0–31.4 kg/m^2^). The median CTDI_vol_ was 4.1 mGy at routine dose and 2.2, 1.6 and 1.0 mGy at reduced dose levels for the 120-kVp acquisitions. For the 100-kVp acquisition, the median CTDI_vol_ was 2.4, 1.3, 1.0 and 0.6 mGy, respectively. The median DLP was 150 (96–169), 84 (53–93), 60 (38–66) and 39 (24–42) mGy ×cm, respectively, resulting in median effective dose levels of 2.2 (1.4–2.4), 1.2 (0.8–1.3), 0.9 (0.5–1.0) and 0.6 (0.3–0.6) mSv.

### Emphysema

**Fig. 2:**
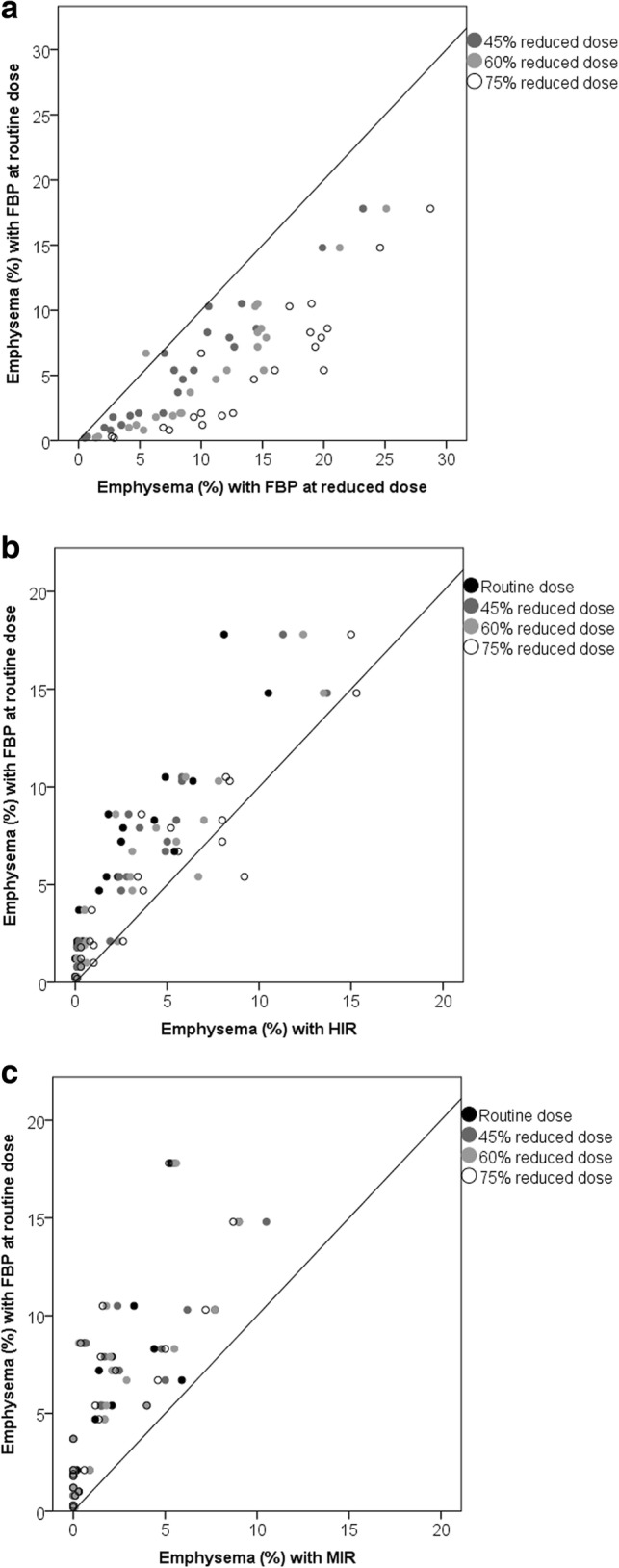
*Scatterplots* of the effect of radiation dose and reconstruction on the percentage emphysema. The *y-axis* displays the percentage emphysema with FBP at routine dose (reference), while the *x-axis* displays the percentage emphysema at reduced dose with FPB (**a**) and with HIR (**b**) and MIR (**c**). Values below the diagonal represent an overestimation of the percentage of emphysema as compared to FBP and routine dose, while values above the diagonal represent an underestimation. *FBP* filtered back projection, *HIR* hybrid iterative reconstruction, *MIR* model-based iterative reconstruction

**Fig. 3:**
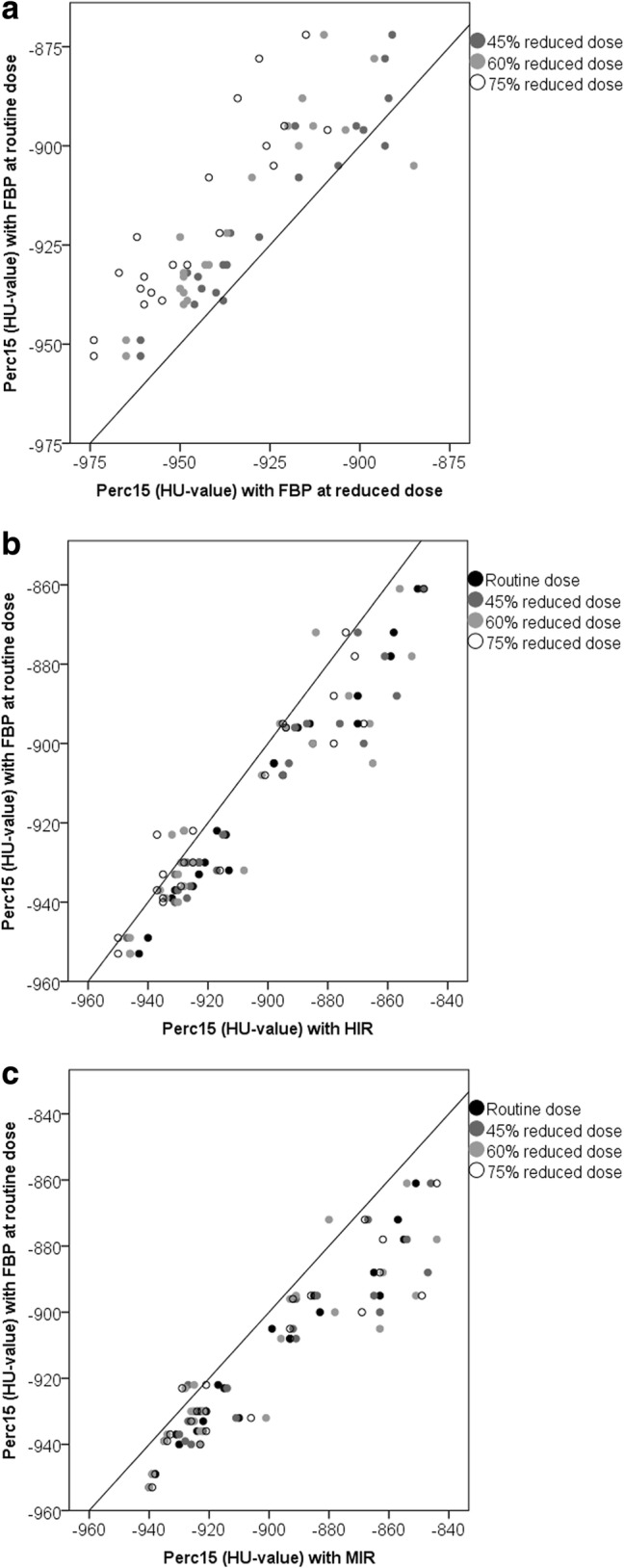
*Scatterplots* of the effect of radiation dose and reconstruction on the perc_15_. The *y-axis* displays the perc_15_ with FBP at routine dose (reference), while the *x-axis* displays the perc_15_ at reduced dose with FBP (**a**) and with HIR (**b**) and MIR (**c**). Values below the diagonal represent a higher HU value compared to the reference, while values above the diagonal represent a lower HU value compared to the reference. *FBP* filtered back projection, *HIR* hybrid iterative reconstruction, *MIR* model-based iterative reconstruction

The percentage of emphysema with FBP at routine dose was 5.1% (1.7–8.4%). FBP at reduced dose resulted in a significant overestimation of the percentage of emphysema, while HIR and MIR resulted in a significant underestimation at all dose levels compared to FBP at routine dose (Table 1, Fig. 2). The perc_15_ measurements resulted in decreased HU values for FBP at reduced dose, while HIR and MIR resulted in significantly increased HU values compared to FBP at routine dose (Table 1, Fig. 3). For the -950 HU threshold, HIR at 75% reduced dose resulted in the highest ICC of 0.92 (0.76–0.97), while the ICC decreased to 0.42 (0.00–0.79) with FBP at 75% reduced dose. Overall, the ICC was better with the perc_15_ method, resulting in a minimum ICC of 0.59 (0.00–0.88) with FBP at 75% reduced dose and a maximum ICC of 0.94 (0.81–0.98) with HIR at 75% reduced dose.

**Table 1:** Percentage of emphysema using the -950 HU threshold and the perc_15_ method at different dose levels reconstructed with FBP, HIR and MIR

	-950 HU (%)	ICC (95% CI)	Perc_15_ (HU-value)	ICC (95%CI)
Routine dose				
FBP	5.1 (1.7–8.4)	NA	-923 (-936 – -895)	NA
HIR	1.5 (0.1–4.5)^a^	0.63 (0.00–0.88)	-914 (-927 – -881)^a^	0.91 (0.00–0.98)
MIR	0.9 (0.0–3.6)^a^	0.50 (0.00–0.80)	-913 (-926 – -879)^a^	0.88 (0.01–0.97)
45% reduced dose				
FBP	8.0 (3.3–12.4)^a^	0.83 (0.00–0.96)	-932 (-944 – -898)^a^	0.93 (0.39–0.98)
HIR	2.5 (0.2–5.1)^a^	0.79 (0.01–0.94)	-916 (-929 – -875)^a^	0.89 (0.28–0.97)
MIR	1.2 (0.0–3.1)^a^	0.51 (0.00–0.81)	-913 (-927 – -867)^a^	0.83 (0.07–0.95)
60% reduced dose				
FBP	10.2 (5.5–14.7)^a^	0.63 (0.00–0.89)	-940 (-949 – -912)^a^	0.77 (0.00–0.94)
HIR	2.7 (0.5–6.2)^a^	0.83 (0.24–0.95)	-917 (-931 – -881)^a^	0.87 (0.62–0.95)
MIR	1.3 (0.0–3.2)^a^	0.49 (0.00–0.79)	-911 (-927 – -874)^a^	0.80 (0.28–0.93)
75% reduced dose				
FBP	14.3 (9.7–19.6)^a^	0.42 (0.00–0.79)	-948 (-961 – -925)^a^	0.59 (0.00–0.88)
HIR	3.5 (0.7–8.1)^a^	0.92 (0.76–0.97)	-921 (-935 – -878)^a^	0.94 (0.81–0.98)
MIR	0.9 (0.0–4.2)^a^	0.47 (0.00–0.78)	-914 (-927 – -869)^a^	0.84 (0.16–0.95)

Values represent the median (interquartile range). The ICC compares with the reference standard, namely FBP at routine dose

^a^Statistically significant difference compared to FBP at routine dose with a Bonferroni corrected *p* value of 0.017

*FBP* filtered back projection, *HIR* hybrid iterative reconstruction, *ICC* intraclass correlation coefficient, *MIR* model-based iterative reconstruction

**Fig. 4:**
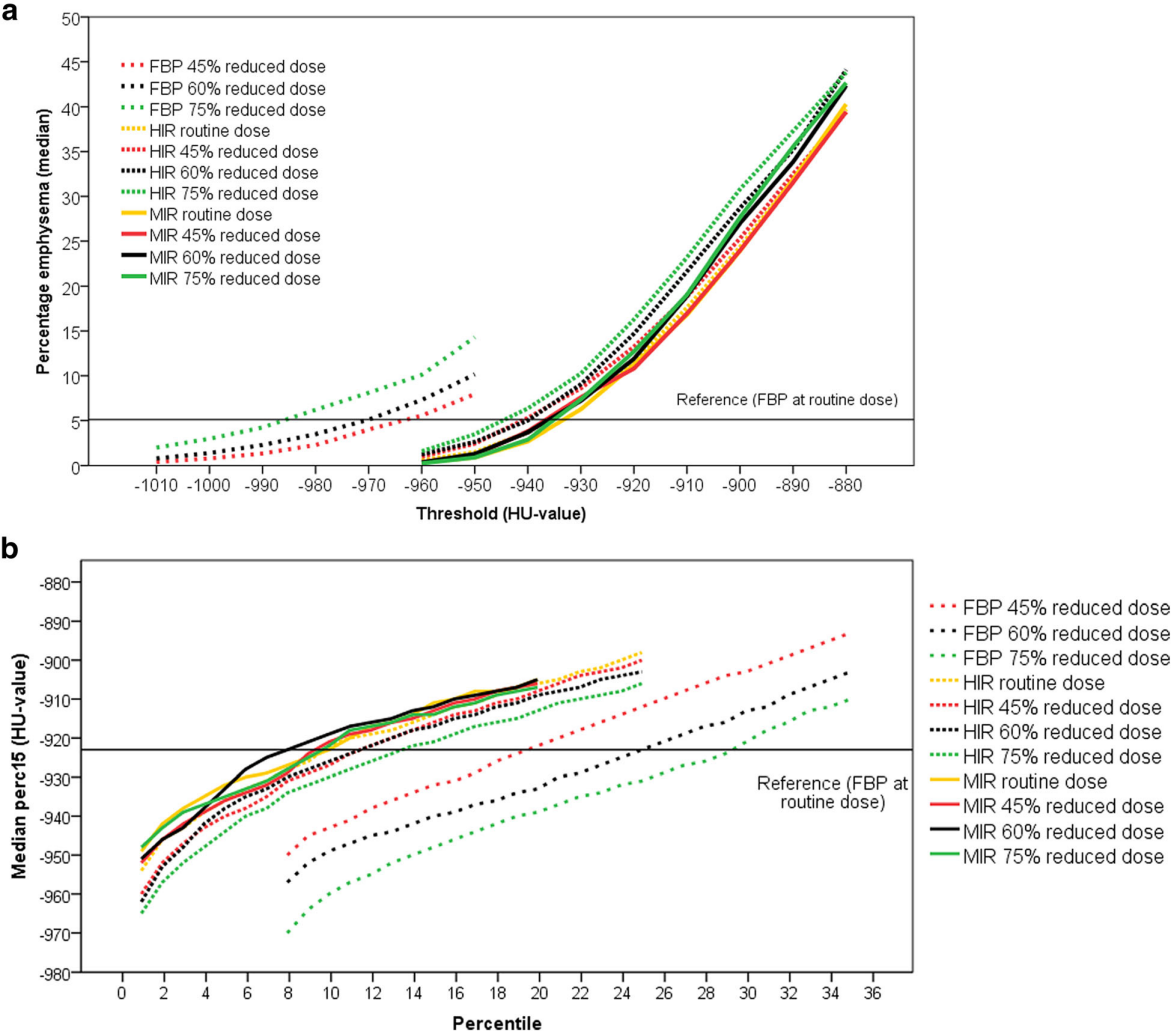
Effect of different thresholds (**a**) and percentiles (**b**) on emphysema quantification. For FBP at reduced dose a lower threshold is more appropriate, while with HIR and MIR a higher HU threshold should be used. With the percentile quantification method, FBP at reduced dose requires a higher percentile while with HIR and MIR a lower percentile should be used to achieve the same results as with FBP at routine dose. *FBP* filtered back projection, *HIR* hybrid iterative reconstruction, *MIR* model-based iterative reconstruction

The effect of using different HU thresholds or percentiles is shown in Fig. 4. Data for each threshold are provided in the supplemental files (Additional file 1: Table S1–S4). With FBP at reduced dose, using a lower threshold of -960 HU, -970 HU and -980 HU at 45%, 60% and 75% reduced dose, respectively, resulted in a percentage of emphysema that was not significantly different from the reference standard. For HIR, a threshold of -930 HU (routine dose) or -940 HU (reduced dose) approximated the percentage emphysema with FBP at routine dose, while this was -930 HU for MIR (all dose levels). Bland-Altman plots are provided in Additional file 2: Figure S1 of the supplemental files. The adapted threshold worked well over the whole range of patients for FBP at reduced dose, while with HIR and MIR there was a trend towards underestimation in patients with a small emphysema percentage and in patients with a higher percentage of emphysema there was an overestimation.

FBP at reduced dose required a higher percentile of 19%, 22% and 26%, respectively, at 45%, 60% and 75% reduced dose to achieve results comparable to the reference standard. With HIR and MIR, a lower percentile was required of 8% (routine dose), 10% (45% reduced dose), 11% (60% reduced dose) or 13% (75% reduced dose) with HIR and 8% (all dose levels) with MIR. Bland–Altman plots are provided in Additional file 2: Figure S2 of the supplemental files. The adapted threshold worked well over the whole range of patients for all reconstructions.

### Image quality

**Fig. 5:**
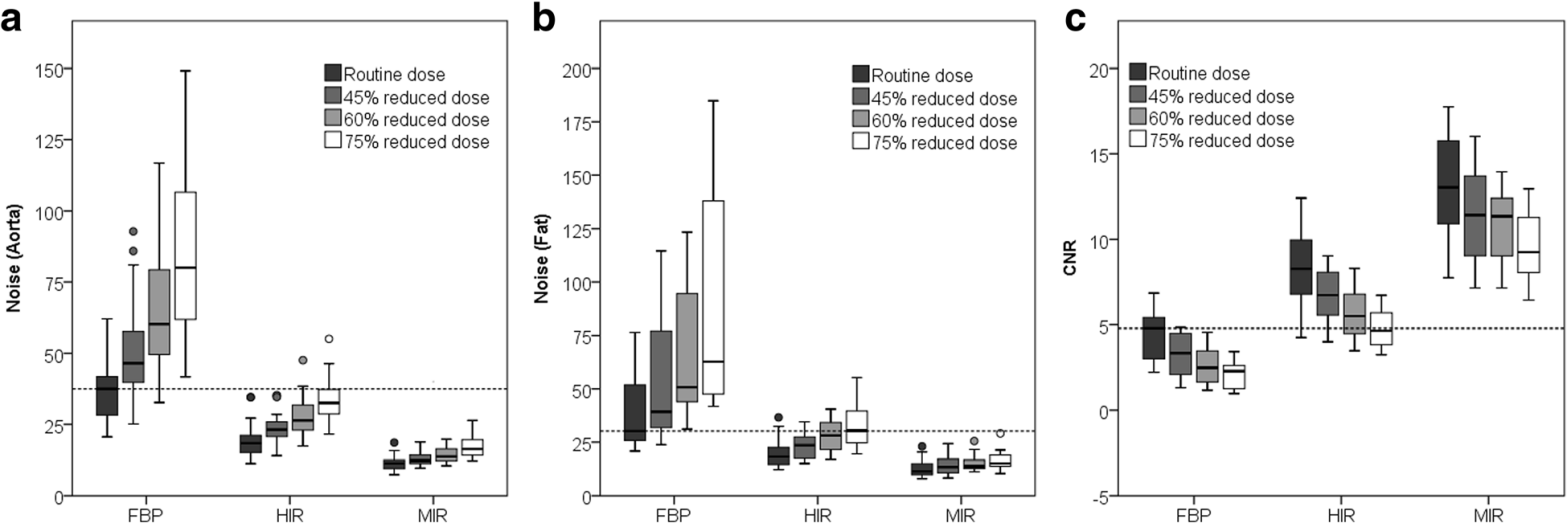
Noise (**a** and **b**) and CNR (**c**) measured at different dose levels with FBP, HIR and MIR. Noise was measured in the aorta (**a**) and subcutaneous fat (**b**). The *dotted line* represents the reference (FBP at routine dose). *CNR* contrast-to-noise ratio, *FBP* filtered back projection, *HIR* hybrid iterative reconstruction, *MIR* model-based iterative reconstruction

Noise and CNR are presented in Fig. 5 and Table 2. Noise increased with FBP at reduced dose levels, while HIR and MIR resulted in reduced noise. Noise was significantly lower with HIR at routine dose, 45% reduced dose and 60% reduced dose compared to the reference standard, while at the lowest dose level noise was comparable to FBP at routine dose. MIR resulted in a significant reduction of noise at all reduced dose levels compared to FBP at routine dose. CNR decreased with FBP at reduced dose levels. HIR and MIR resulted in comparable or improved CNR at all reduced dose levels.

**Table 2:** Noise and CNR at different dose levels with FBP, HIR and MIR

	Noise (aorta)	Noise (fat)	CNR
Routine dose			
FBP	37.5 (28.1–42.8)	30.2 (25.4–52.0)	4.8 (3.0–5.5)
HIR	18.4 (15.0–22.2)^a^	18.2 (14.6–23.3)^a^	8.3 (6.7–10.1)^a^
MIR	11.4 (9.5–12.5)^a^	11.5 (9.8–15.2)^a^	13.0 (10.7–15.9)^a^
45% reduced dose			
FBP	46.6 (38.9–58.4)^a^	39.3 (31.6–78.9)^a^	3.3 (2.1–4.5)^a^
HIR	23.2 (20.5–26.5)^a^	23.5 (17.5–27.8)^a^	6.7 (5.5–8.1)^a^
MIR	12.3 (11.3–14.4)^a^	13.5 (10.7–17.2)^a^	11.4 (9.0–13.7)^a^
60% reduced dose			
FBP	60.2 (47.8–81.4)^a^	50.8 (42.5–94.6)^a^	2.5 (1.7–3.5)^a^
HIR	26.4 (22.9–31.8)^a^	28.1 (21.4–34.7)^a^	5.5 (4.4–6.8)^a^
MIR	13.7 (12.2–16.5)^a^	13.9 (12.7–17.2)^a^	11.4 (8.9–12.4)^a^
75% reduced dose			
FBP	80.0 (61.4–108.5)^a^	62.8 (47.5–142.6)^a^	2.3 (1.2–2.7)^a^
HIR	32.6 (28.5–37.8)	30.4 (24.7–39.6)	4.7 (3.7–5.7)
MIR	16.4 (14.1–19.7)^a^	15.1 (13.8–19.1)^a^	9.2 (7.9–11.3)^a^

Values are presented as median (interquartile range)

^a^Statistically significant difference compared to FBP at routine dose with a Bonferroni corrected *p* value of 0.017

*CNR* contrast-to-noise ratio, *FBP* filtered back projection, *HIR* hybrid iterative reconstruction, *MIR* model-based iterative reconstruction

## Discussion

This study shows the effect of different reconstruction techniques at four decreasing radiation dose levels. While FBP resulted in an overestimation of emphysema on CT at reduced dose, both HIR and MIR resulted in an underestimation of the amount of emphysema compared to reconstruction of the images with FBP at routine dose. Furthermore, we showed that by using different thresholds or percentages in HIR and MIR, it was possible to derive results comparable to FBP at routine dose.

There are two commonly used measures to quantify emphysema on CT based on densitometry, namely the density at the 15th percentile of the attenuation curve and the percentage of voxels below -950 HU. Previous studies indicated that those thresholds show the strongest correlation with microscopic and macroscopic emphysema findings in studies using FBP [8, 9]. The 15th percentile and the -950 HU thresholds are widely used; however, different thresholds have been applied in the literature [8, 9, 20, 21]. Several other studies have investigated the effect of dose and image reconstruction on pulmonary emphysema quantification. Schilham et al. [22] compared a clinical routine dose CT acquisition with a low dose acquisition in 25 patients. A post-processing filter was used to reduce the amount of noise in the low dose images and three different thresholds (-950, -930 and -910 HU) were used to quantify emphysema. The application of the filter resulted in a reduction of the effect of noise on the emphysema percentage. A different study by Mets et al. [23] in 75 patients who underwent a routine dose CT acquisition reconstructed with FBP and HIR reported an underestimation of the amount of emphysema with HIR when the cut-off was not adjusted. In a study by Nishio et al. [24], the application of IR at reduced dose improved the agreement in emphysema quantification with routine dose FBP. Three studies comparing a routine dose acquisition with a low-dose acquisition in the same patient all reported an overestimation with low-dose FBP while IR resulted in an underestimation [25–27]. Messerli et al. [27] reduced the radiation dose to chest x-ray equivalent dose levels of 0.14 mSv; at this dose level, HIR resulted in a similar emphysema measurement as FBP at routine dose (1.7 mSv). Similar results were found in the study by Nishio et al. [28]. Therefore, by carefully selecting the dose reduction level, emphysema overestimation can be compensated for by using IR, since the latter results in reduced emphysema with CT quantification. To our best knowledge, only the study by Choo et al. [15] investigated the effects of both HIR and MIR. No dose reduction was used and they reported that MIR resulted in a larger underestimation than HIR compared to FBP, which is comparable to the results of the current study.

The effect of reconstruction technique and radiation dose can be explained by the density histogram. IR algorithms result in a different density distribution, which subsequently affects emphysema quantification. Due to the noise reduction with IR, the extremes of the attenuation distribution are affected [23], leading to a smaller peak in the density histogram. Dose reduction, on the other hand, results in increased image noise, leading to a broadening of the density histogram [29].

In the current study, FBP at routine dose was used as the reference standard. However, ideally a pathological reference standard should be applied or a realistic phantom to determine what is closest to the truth and if thresholds should be adapted. It is important to be aware that differences in emphysema quantification can occur and to keep the radiation dose and reconstruction algorithm constant in longitudinal follow-up studies.

Although this within-patient study systematically assessed the effect of dose and reconstruction on emphysema quantification, there are several limitations. First, the patients included in this study had a low amount of emphysema. Second, the sample size was relatively low; however, due to the within-patients study design, the statistical power of the study was increased. Although we showed that adapting the commonly used thresholds can prevent underestimation of emphysema with IR, the sample size was too small to give a clear recommendation about the optimal threshold. Third, one software package and IR algorithms from only one vendor were studied and results may differ for other packages and other vendors. Fourth, only an inspiratory chest CT was acquired; therefore, air-trapping could not be studied. Fifth, the effect of slice thickness and reconstruction kernel were not investigated in the current article. Gierada et al. [30] investigated the effect of reconstruction kernel and slice thickness and reported that patients with 10–30% emphysema are most sensitive for the effect of kernel and slice thickness, while lower emphysema percentages (such as in the current study) resulted in more stable measurements.

In conclusion, as compared to FBP at routine dose, both HIR and MIR result in an underestimation of CT emphysema at routine dose and reduced dose while FBP results in an overestimation at reduced dose. This can potentially be solved by using adapted thresholds.

## Additional files

Additional file 1: **Table S1.** Effect of different HU thresholds on emphysema quantification. Values represent the median [interquartile range] percentage emphysema at each dose level with FBP, HIR and MIR. *FBP* filtered back projection, *HIR* hybrid iterative reconstruction; *MIR* model-based iterative reconstruction, *NA* not applicable. **Table S2.** Effect of different percentiles on emphysema quantification. Values represent the median [interquartile range] HU value at each dose level with FBP. *FBP* filtered back projection; *NA* not applicable. **Table S3.** Effect of different percentiles on emphysema quantification. Values represent the median [interquartile range] HU value at each dose level with HIR. *HIR* hybrid iterative reconstruction; *NA* not applicable. **Table S4.** Effect of different percentiles on emphysema quantification. Values represent the median [interquartile range] HU value at each dose level with MIR. *MIR* model-based iterative reconstruction. (DOCX 35 kb)

Additional file 2: **Figure S1.** Bland–Altman plots for the differences in percentage emphysema when comparing the optimal adapted threshold at each dose level to FBP at routine dose using a − 950 HU threshold. The continuous line represents the mean difference to the reference standard while the dotted lines represent the upper and lower limits of agreement (95% limits of agreement). FBP filtered back projection, HIR hybrid iterative reconstruction; MIR model-based iterative reconstruction. **Figure S2.** Bland–Altman plots for the differences in HU value when comparing the optimal adapted threshold at each dose level with FBP at routine dose using the perc15 method. The continuous line represents the mean difference to the reference standard while the dotted lines represent the upper and lower limits of agreement (95% limits of agreement). FBP filtered back projection; HIR hybrid iterative reconstruction; MIR model-based iterative reconstruction. (PDF 578 kb)

## Abbreviation

CNR: Contrast-to-noise ratio
COPD: Obstructive pulmonary disease
CT: Computed tomography
CTDIvol: Volume CT dose index
DLP: Dose-length product
FBP: Filtered back projection
HIR: Hybrid IR
ICC: Intraclass correlation coefficient
IR: Iterative reconstruction
MIR: Model-based IR
