# Supplementary material for: Emphysema quantification using chest CT: influence of radiation dose reduction and reconstruction technique
Source: Eur Radiol Exp. 2018 Nov 7;2:30. doi: 10.1186/s41747-018-0064-3 (PMC6220000; doi:10.1186/s41747-018-0064-3)
Supplement: Supplementary file 1 — Table S1. Effect of different HU thresholds on emphysema quantification. Values represent the median [interquartile range] percentage emphysema at each dose level with FBP, HIR and MIR. FBP filtered back projection, HIR hybrid iterative reconstruction; MIR model-based iterative reconstruction, NA not applicable. Table S2. Effect of different percentiles on emphysema quantification. Values represent the median [interquartile range] HU value at each dose level with FBP. FBP filtered back projection; NA not applicable. Table S3. Effect of different percentiles on emphysema quantification. Values represent the median [interquartile range] HU value at each dose level with HIR. HIR hybrid iterative reconstruction; NA not applicable. Table S4. Effect of different percentiles on emphysema quantification. Values represent the median [interquartile range] HU value at each dose level with MIR. MIR model-based iterative reconstruction. (DOCX 35 kb) [file 41747_2018_64_MOESM1_ESM.docx]

**Additional file**

**Table S1** – Effect of different HU-thresholds on emphysema quantification. Values represent the median [interquartiles] percentage emphysema at each dose level with FBP, HIR and MIR. *FBP Filtered Back Projection; HIR Hybrid Iterative Reconstruction; MIR Model-based Iterative Reconstruction; NA Not Applicable*

|  | **FBP** | | | | **HIR** | | | | **MIR** | | | |
| --- | --- | --- | --- | --- | --- | --- | --- | --- | --- | --- | --- | --- |
|  | Routine dose | 45% reduced dose | 60% reduced dose | 75% reduced dose | Routine dose | 45% reduced dose | 60% reduced dose | 75% reduced dose | Routine dose | 45% reduced dose | 60% reduced dose | 75% reduced dose |
| **-880HU** | NA | | | | 39.8 [17.3 – 59.6] | 39.4 [12.6 – 56.4] | 44.1 [16.3 – 59.8] | 43.8 [14.4 – 55.9] | 40.3 [15.4 – 61.5] | 39.5  [8.1 – 58.1] | 42.4 [13.3 – 58.3] | 42.7  [9.3 – 57.8] |
| **-890HU** |  |  |  |  | 31.9 [10.9 – 50.0] | 32.3  [8.7 – 49.9] | 35.2 [11.8 – 49.0] | 37.2 [10.6 – 48.5] | 31.9  [8.8 – 52.3] | 31.6  [4.8 – 50.2] | 33.9  [9.2 – 49.4] | 35.6  [5.6 – 48.8] |
| **-900HU** |  |  |  |  | 24.4  [6.2 – 41.0] | 25.3  [5.8 – 41.1] | 28.7  [8.1 – 41.2] | 30.8  [7.6 – 41.4] | 23.9  [4.4 – 40.8] | 24.0  [2.6 – 41.1] | 27.0  [5.1 – 40.0] | 27.7  [3.0 – 40.1] |
| **-910HU** |  |  |  |  | 17.6  [2.7 – 30.7] | 18.9  [3.2 – 32.1] | 21.7  [4.8 – 32.2] | 23.3  [5.3 – 33.2] | 16.8  [1.9 – 31.2] | 17.0  [1.3 – 31.2] | 18.9  [2.6 – 32.1] | 19.1  [1.6 – 30.7] |
| **-920HU** |  |  |  |  | 11.6  [1.3 – 20.5] | 13.3  [1.3 – 22.5] | 14.7  [2.7 – 23.9] | 16.3  [2.9 – 25.1] | 11.3  [0.8 – 20.5] | 10.8  [0.6 – 21.4] | 11.9  [1.2 – 22.7] | 12.7  [0.8 – 21.1] |
| **-930HU** |  |  |  |  | 7.3  [0.6 – 12.4] | 8.6  [0.6 – 14.1] | 9.1  [1.5 – 15.8] | 10.3  [1.8 – 18.3] | 6.3  [0.2 – 11.7] | 7.6  [0.2 – 12.6] | 7.2  [0.5 – 12.6] | 7.4  [0.3 – 13.0] |
| **-940HU** |  |  |  |  | 3.7  [0.2 – 7.5] | 5.3  [0.4 – 9.3] | 5.0  [0.9 – 10.2] | 6.4  1.2 – 12.7] | 2.7  [0.1 – 7.5] | 3.8  [0.1 – 6.3] | 3.7  [0.1 – 6.2] | 2.9  [0.1 – 6.7] |
| **-950HU** | 5.1  [1.7 – 8.4] | 8.0  [3.3 – 12.4] | 10.2  [5.5 – 14.7] | 14.3  [9.7 – 19.6] | 1.5  [0.1 – 4.5] | 2.5  [0.2 – 5.1] | 2.7  [0.5 – 6.2] | 3.5  [0.7 – 8.1] | 0.9  [0.0 – 3.6] | 1.2  [0.0 – 3.1] | 1.3  [0.0 – 3.2] | 0.9  [0.0 – 4.2] |
| **-960HU** | NA | 5.6  [2.5 – 8.7] | 7.3  [4.3 – 11.0] | 10.1  [7.0 – 15.4] | 0.6  [0.0 – 1.9] | 1.0  [0.1 – 2.9] | 1.3  [0.2 – 3.4] | 1.6  [0.3 – 4.8] | 0.3  [0.0 – 1.1] | 0.4  [0.0 – 0.9] | 0.4  [0.0 – 1.5] | 0.3  [0.0 – 1.4] |
| **-970HU** |  | 4.0  [1.7 – 5.8] | 5.1  [2.7 – 7.9] | 8.1  [5.2 – 11.9] | NA | | | | | | | |
| **-980HU** |  | 2.3  [0.9 – 3.8] | 3.5  [2.0 – 5.4] | 6.2  [3.9 – 8.9] |  |  |  |  |  |  |  |  |
| **-990HU** |  | 1.4  [0.6 – 2.5] | 2.3  [1.4 – 3.6] | 4.2  [2.6 – 6.4] |  |  |  |  |  |  |  |  |
| **-1000HU** |  | 0.8  [0.3 – 1.7] | 1.4  [0.8 – 2.4] | 3.0  [1.7 – 4.3] |  |  |  |  |  |  |  |  |
| **-1010HU** |  | 0.4  [0.2 – 0.9] | 0.8  [0.5 – 1.4] | 2.0  [1.1 – 2.8] |  |  |  |  |  |  |  |  |

**Table S2** – Effect of different percentiles on emphysema quantification. Values represent the median [interquartiles] HU value at each dose level with FBP. *FBP Filtered Back Projection; NA Not Applicable*

|  | **FBP** | | | |  | **FBP** | | | |
| --- | --- | --- | --- | --- | --- | --- | --- | --- | --- |
|  | Routine dose | 45% reduced dose | 60% reduced dose | 75% reduced dose |  | Routine dose | 45% reduced dose | 60% reduced dose | 75% reduced dose |
| **Perc8** | NA | -950 [-961 – -920] | -957 [-969 – -934] | -970 [-983 – -956] | **Perc22** | NA | -918 [-931 – -878] | -929 [-934 – -892] | -935 [-944 – -903] |
| **Perc9** |  | -945 [-958 – -915] | -952 [-966 – -931] | -964 [-979 – -952] | **Perc23** |  | -916 [-929 – -874] | -927 [-932 – -889] | -934 [-942 – -900] |
| **Perc10** |  | -943 [-955 – -912] | -949 [-963 – -927] | -960 [-976 – -948] | **Perc24** |  | -914 [-927 – -782] | -925 [-930 – -886] | -932 [-940 – -897] |
| **Perc11** |  | -941 [-953 – -909] | -947 [-959 – -923] | -957 [-972 – -943] | **Perc25** |  | -912 [-925 – -870] | -923 [-928 – -884] | -931 [-938 – -893] |
| **Perc12** |  | -938 [-950 – -904] | -945 [-956 – -920] | -955 [-970 – -939] | **Perc26** |  | -910 [-924 – -867] | -921 [-927 – -881] | -929 [-936 – -890] |
| **Perc13** |  | -936 [-948 – -902] | -944 [-954 – -918] | -952 [-967 – -934] | **Perc27** |  | -908 [-923 – -865] | -919 [-925 – -879] | -927 [-934 – -888] |
| **Perc14** |  | -934 [-946 – -899] | -942 [-951 – -915] | -950 [-964 – -930] | **Perc28** |  | -906 [-921 – -863] | -917 [-924 – -877] | -926 [-932 – -885] |
| **Perc15** | -923 [-936 – -895] | -932 [-944 – -898] | -940 [-949 – -912] | -948 [-961 – -925] | **Perc39** |  | -904 [-920 – -860] | -916 [-922 – -874] | -924 [-929 – -882] |
| **Perc16** | NA | -931 [-942 – -896] | -939 [-946 – -910] | -946 [-958 – -921] | **Perc30** |  | -903 [-919 – -858] | -913 [-921 – -872] | -921 [-927 – -879] |
| **Perc17** |  | -929 [-940 – -894] | -937 [-944 – -907] | -944 [-956 – -918] | **Perc31** |  | -901 [-917 – -856] | -912 [-920 – -870] | -918 [-925 – -876] |
| **Perc18** |  | -926 [-938 – -891] | -936 [-942 – -904] | -942 [-953 – -915] | **Perc32** |  | -899 [-916 – -854] | -909 [-918 – -867] | -916 [-923 – -874] |
| **Perc19** |  | -924 [-936 – -888] | -934 [-940 – -901] | -940 [-951 – -912] | **Perc33** |  | -897 [-914 – -853] | -907 [-917 – -865] | -913 [-921 – -871] |
| **Perc20** |  | -922 [-935 – -884] | -933 [-938 – -897] | -939 [-949 – -909] | **Perc34** |  | -895 [-913 – -851] | -905 [-916 – -862] | -912 [-919 – -869] |
| **Perc21** |  | -920 [-933 – -881] | -930 [-936 – -895] | -937 [-946 – -907] | **Perc35** |  | -849 [-912 – -849] | -903 [-914 – -860] | -910 [-917 – -866] |

|  | **HIR** | | | |  | **HIR** | | | |
| --- | --- | --- | --- | --- | --- | --- | --- | --- | --- |
|  | Routine dose | 45% reduced dose | 60% reduced dose | 75% reduced dose |  | Routine dose | 45% reduced dose | 60% reduced dose | 75% reduced dose |
| **Perc1** | -954 [-965 – -923] | -960 [-974 – -923] | -962 [-979 – -937] | -965 [-987 – -943] | **Perc14** | -916 [-928 – -883] | -918 [-930 – -877] | -918 [-933 – -884] | -922 [-937 – -881] |
| **Perc2** | -946 [-959 – -914] | -952 [-965 – -914] | -953 [-967 – -925] | -957 [-976 – -928] | **Perc15** | -914 [-927 – -881] | -916 [-929 – -875] | -917 [-931 – -881] | -921 [-935 – -878] |
| **Perc3** | -943 [-955 – -908] | -947 [-959 – -910] | -948 [-961 – -918] | -952 [-970 – -919] | **Perc16** | -912 [-925 – -880] | -914 [-928 – -873] | -915 [-930 – -879] | -919 [-933 – -876] |
| **Perc4** | -939 [-951 – -905] | -943 [-955 – -906] | -942 [-957 – -913] | -948 [-963 – -914] | **Perc17** | -911 [-924 – -879] | -913 [-926 – -871] | -914 [-928 – -877] | -917 [-931 – -874] |
| **Perc5** | -936 [-948 – -902] | -940 [-950 – -903] | -938 [-953 – -909] | -944 [-959 – -910] | **Perc18** | -909 [-923 – -877] | -911 [-925 – -869] | -912 [-926 – -876] | -916 [-930 – -872] |
| **Perc6** | -933 [-945 – -899] | -938 [-947 – -899] | -935 [-950 – -905] | -940 [-955 – -906] | **Perc19** | -907 [-921 – -876] | -910 [-924 – -867] | -911 [-925 – -874] | -915 [-928 – -871] |
| **Perc7** | -931 [-940 – -897] | -935 [-945 – -895] | -933 [-947 – -902] | -938 [-925 – -902] | **Perc20** | -906 [-920 – -875] | -908 [-923 – -865] | -909 [-924 – -872] | -913 [-927 – -868] |
| **Perc8** | -928 [-927 – -894] | -931 [-942 – -891] | -930 [-945 – -900] | -934 [-950 – -898] | **Perc21** | -905 [-919 – -872] | -906 [-922 – -864] | -908 [-923 – -871] | -911 [-925 – -866] |
| **Perc9** | -926 [-935 – -892] | -929 [-940 – -888] | -928 [-942 – -897] | -932 [-947 – -895] | **Perc22** | -903 [-918 – -870] | -904 [-920 – -862] | -907 [-921 – -869] | -910 [-924 – -865] |
| **Perc10** | -922 [-933 – -890] | -927 [-938 – -886] | -926 [-940 – -894] | -930 [-945 – -891] | **Perc23** | -902 [-917 – -867] | -903 [-919 – -860] | -905 [-920 – -867] | -909 [-922 – -863] |
| **Perc11** | -920 [-932 – -888] | -924 [-936 – -883] | -924 [-938 – -891] | -928 [-943 – -888] | **Perc24** | -900 [-916 – -864] | -902 [-918 – -859] | -904 [-919 – -865] | -908 [-921 – -862] |
| **Perc12** | -919 [-930 – -887] | -922 [-934 – -881] | -922 [-936 – -888] | -926 [-941 – -886] | **Perc25** | -899 [-915 – -862] | -900 [-917 – -857] | -903 [-918 – -864] | -906 [-920 – -860] |
| **Perc13** | -918 [-929 – -885] | -920 [-932 – -879] | -920 [-934 – -886] | -924 [-939 – -883] |  | | | | |

**Table S3** – Effect of different percentiles on emphysema quantification. Values represent the median [interquartiles] HU value at each dose level with HIR. *HIR Hybrid Iterative Reconstruction; NA Not Applicable*

|  | **MIR** | | | |  | **MIR** | | | |
| --- | --- | --- | --- | --- | --- | --- | --- | --- | --- |
|  | Routine dose | 45% reduced dose | 60% reduced dose | 75% reduced dose |  | Routine dose | 45% reduced dose | 60% reduced dose | 75% reduced dose |
| **Perc1** | -949 [-960 – -916] | -952 [-958 – -914] | -951 [-962 – -922] | -948 [-962 – -916] | **Perc11** | -920 [-930 – -885] | -919 [-931 – -873] | -917 [-932 – -885] | -918 [-933 – -876] |
| **Perc2** | -942 [-955 – -908] | -946 [-952 – -903] | -946 [-957 – -913] | -943 [-957 – -906] | **Perc12** | -917 [-929 – -883] | -918 [-930 – -871] | -916 [-931 – -881] | -917 [-931 – -874] |
| **Perc3** | -938 [-951 – -903] | -942 [-950 – -897] | -943 [-949 – -908] | -939 [-953 – -900] | **Perc13** | -916 [-928 – -882] | -916 [-929 – -870] | -915 [-929 – -879] | -916 [-930 – -872] |
| **Perc4** | -935 [-948 – -899] | -939 [-947 – -893] | -938 [-945 – -904] | -937 [-951 – -895] | **Perc14** | -914 [-927 – -880] | -915 [-928 – -868] | -913 [-928 – -876] | -914 [-930 – -872] |
| **Perc5** | -932 [-946 – -896] | -936 [-944 – -889] | -933 [-943 – -900] | -935 [-947 – -891] | **Perc15** | -913 [-926 – -879] | -913 [-927 – -867] | -912 [-927 – -874] | -914 [-929 – -871] |
| **Perc6** | -930 [-944 – -893] | -934 [-941 – -886] | -928 [-940 – -897] | -933 [-941 – -888] | **Perc16** | -911 [-924 – -877] | -911 [-926 – -865] | -910 [-926 – -872] | -912 [-926 – -868] |
| **Perc7** | -929 [-941 – -891] | -932 [-938 – -883] | -925 [-938 – -894] | -931 [-939 – -886] | **Perc17** | -910 [-923 – -876] | -910 [-924 – -863] | -909 [-925 – -871] | -911 [-925 – -866] |
| **Perc8** | -927 [-937 – -889] | -929 [-936 – -880] | -923 [-936 – -892] | -928 [-937 – -883] | **Perc18** | -908 [-922 – -875] | -908 [-923 – -862] | -908 [-924 – -870] | -909 [-924 – -865] |
| **Perc9** | -925 [-933 – -888] | -924 [-934 – -878] | -921 [-934 – -890] | -925 [-936 – -880] | **Perc19** | -907 [-921 – -873] | -907 [-922 – -860] | -907 [-922 – -869] | -908 [-922 – -863] |
| **Perc10** | -923 [-932 – -886] | -921 [-933 – -875] | -919 [-933 – -888] | -922 [-934 – -878] | **Perc20** | -905 [-920 – -872] | -906 [-921 – -859] | -905 [-921 – -867] | -907 [-921 – -862] |

**Table S4** – Effect of different percentiles on emphysema quantification. Values represent the median [interquartiles] HU value at each dose level with MIR. *MIR Model-based Iterative Reconstruction*
