# Supplementary material for: Emphysema quantification using chest CT: influence of radiation dose reduction and reconstruction technique
Source: Eur Radiol Exp. 2018 Nov 7;2:30. doi: 10.1186/s41747-018-0064-3 (PMC6220000; doi:10.1186/s41747-018-0064-3)

Figure S1 - Bland-Altman plots for the differences in percentage emphysema when comparing the optimal adapted threshold at each dose level to FBP at routine dose using a -950 HU threshold. The continuous line represents the mean difference to the reference standard while the dotted lines represent the upper and lower limits of agreement (95% limits of agreement). *FBP Filtered Back Projection; HIR Hybrid Iterative Reconstruction; MIR Model-based Iterative Reconstruction*

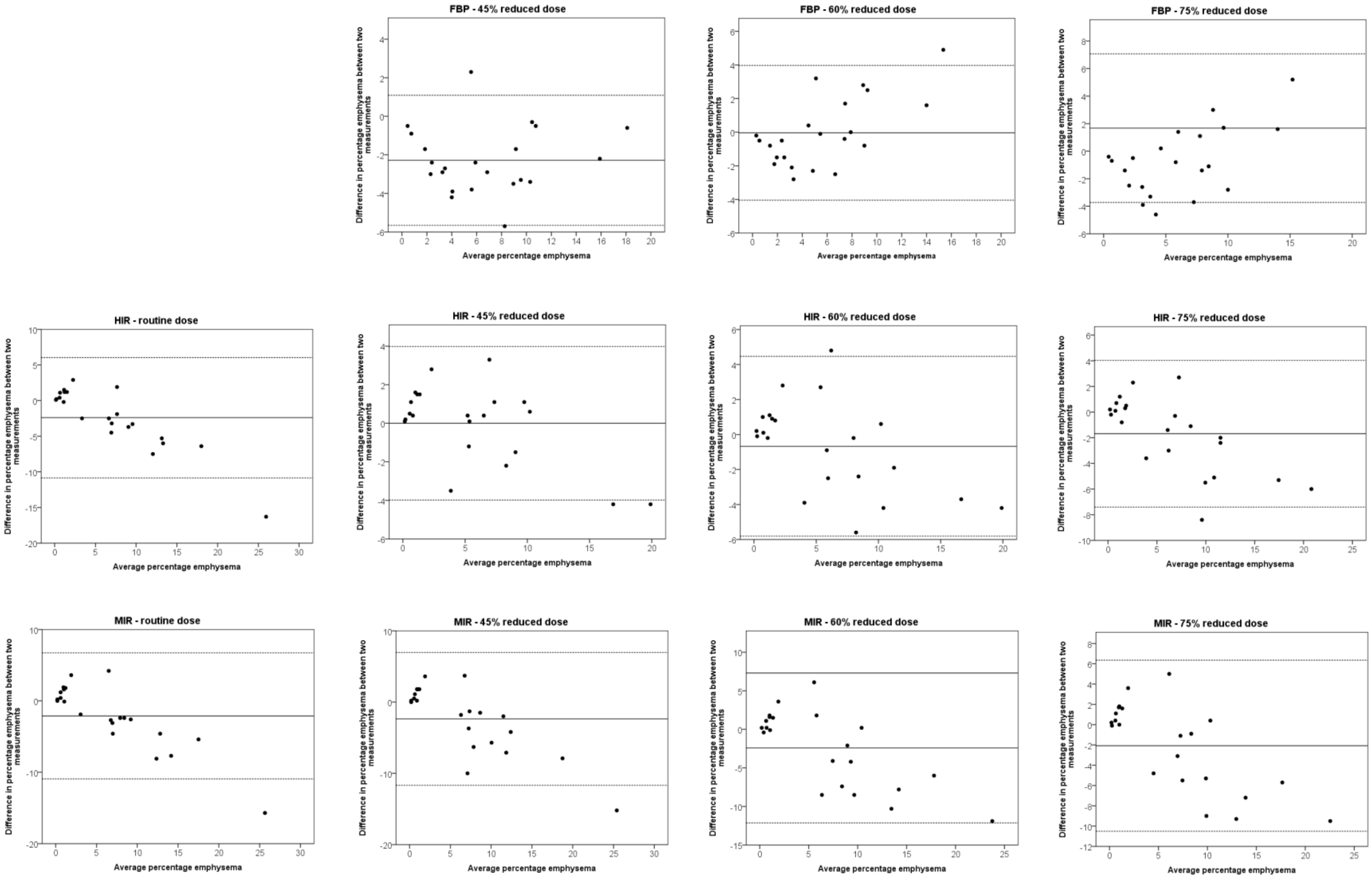

Figure S2 - Bland-Altman plots for the differences in HU-value when comparing the optimal adapted threshold at each dose level with FBP at routine dose using the $perc_{15}$ method. The continuous line represents the mean difference to the reference standard while the dotted lines represent the upper and lower limits of agreement (95% limits of agreement). *FBP Filtered Back Projection; HIR Hybrid Iterative Reconstruction; MIR Model-based Iterative Reconstruction*

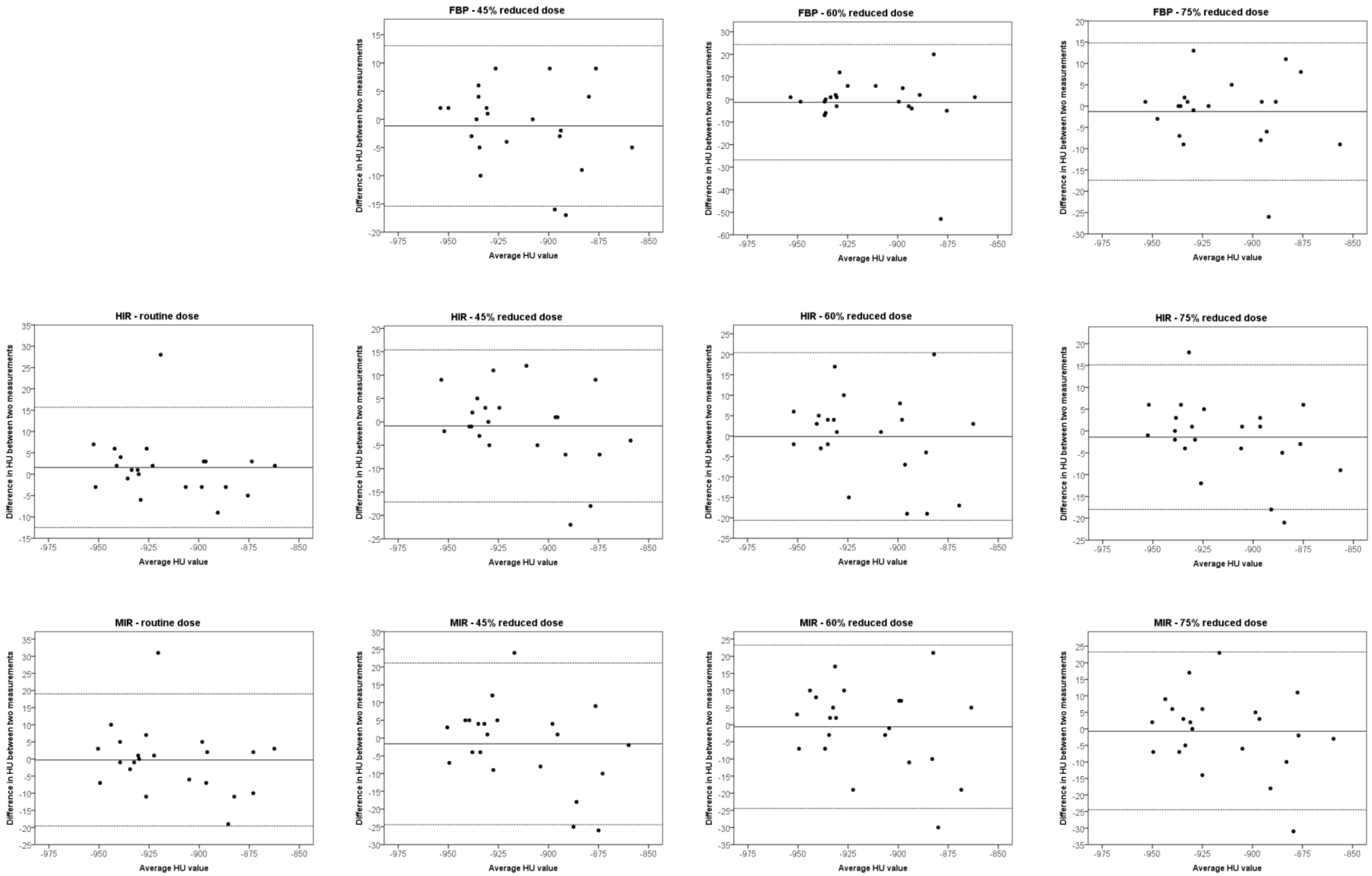

Supplement: Supplementary file 2 — Figure S1. Bland–Altman plots for the differences in percentage emphysema when comparing the optimal adapted threshold at each dose level to FBP at routine dose using a − 950 HU threshold. The continuous line represents the mean difference to the reference standard while the dotted lines represent the upper and lower limits of agreement (95% limits of agreement). FBP filtered back projection, HIR hybrid iterative reconstruction; MIR model-based iterative reconstruction. Figure S2. Bland–Altman plots for the differences in HU value when comparing the optimal adapted threshold at each dose level with FBP at routine dose using the perc15 method. The continuous line represents the mean difference to the reference standard while the dotted lines represent the upper and lower limits of agreement (95% limits of agreement). FBP filtered back projection; HIR hybrid iterative reconstruction; MIR model-based iterative reconstruction. (PDF 578 kb) [file 41747_2018_64_MOESM2_ESM.pdf]
